# Hypovolemic shock due to massive subcutaneous hemorrhage in a patient with musculocontractural Ehlers-Danlos syndrome (mcEDS)

**DOI:** 10.1007/s00508-025-02577-9

**Published:** 2025-08-04

**Authors:** R. Laggner, S. Payr, L. Adam, D. Baron, L. Zak

**Affiliations:** 1 Department of Orthopaedics and Trauma Surgery, Division of Trauma Surgery, Medical University of Vienna, 1090 Vienna, Austria; 2 Department of Anaesthesia, Intensive Care Medicine and Pain Medicine, Medical University of Vienna, Vienna, Austria

**Keywords:** chst14, Musculocontractural ehlers–danlos syndrome, Tissue disorder, Subcutaneous hemorrhage

## Abstract

Musculocontractural Ehlers-Danlos syndrome (mcEDS) is a rare autosomal recessive connective tissue disorder characterized by fragility of skin, vasculature and musculoskeletal structures. We report a case of a young male with CHST14(Carbohydrate sulfotransferase 14)-related mcEDS who developed a massive subcutaneous hematoma following minor trauma, necessitating surgical evacuation. This case highlights the potential for life-threatening bleeding complications in mcEDS and underscores the importance of early recognition and multidisciplinary management.

## Introduction

Ehlers-Danlos syndromes (EDS) are a group of hereditary connective tissue disorders marked by joint hypermobility, skin hyperextensibility, and tissue fragility. The musculocontractural type (mcEDS) is caused by biallelic mutations in CHST14, leading to dermatan sulfate biosynthesis defects and impaired extracellular matrix integrity [1, 2]. Although vascular complications are more prominent in the vascular subtype of EDS, mcEDS can also involve severe bleeding, especially after trauma, due to vessel and soft tissue fragility [3, 4].

## Case presentation

We report a 26-year-old male with a genetically confirmed diagnosis of musculocontractural Ehlers-Danlos syndrome (mcEDS), also referred to as autosomal recessive CHST14 deficiency (ICD-10: Q79.6, ORPHA:2953). His medical history was significant for:

- severe bleeding tendency, with hemorrhage-relevant and painful hematomas frequently requiring surgical evacuation. He had undergone more than 90 general anesthesia procedures for hematoma evacuation and vacuum-assisted closure (VAC) therapy,
- partial amputation of two and one half toes following minor trauma and subsequent massive hematoma formation,
- connective tissue fragility, including joint subluxations, malformations and vascular fragility,
- restrictive pulmonary function impairment,
- grade II mitral valve insufficiency, associated with a dysplastic mitral valve and prolapse of the anterior leaflet,
- congenital anomalies with excellent cognitive development.

At clinical presentation in the trauma outpatient department at 10 p.m. the patient reported a minor trauma occurring 2h before admission, involving a backpack that had fallen onto his lower back while he was putting it on. On admission he was hemodynamically stable and exhibited no immediate signs of clinical deterioration. Clinical examination revealed a soft fluctuant swelling over the lumbar region, measuring approximately 20 × 10 cm.

**Fig. 1:**
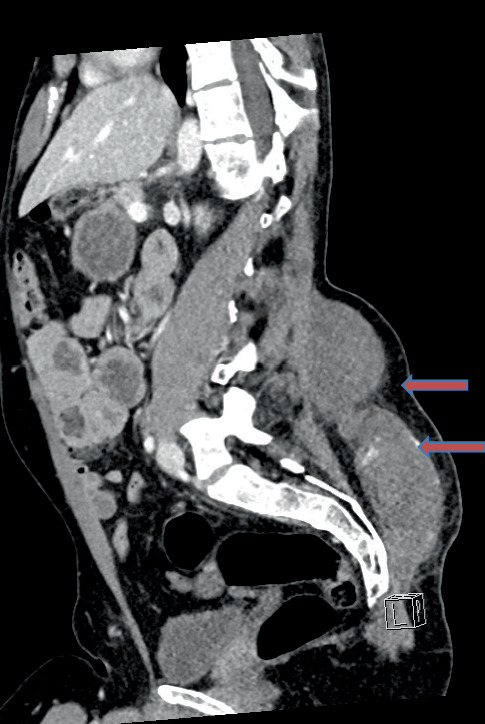
Contrast-enhanced sagittal CT scan of the spine and pelvis on admission, demonstrating a large subcutaneous hematoma in the lumbar region. The hematoma extends cranially from the lumbosacral junction and is associated with multiple sites of contrast extravasation (white areas), indicating ongoing active arterial bleeding

Computed tomography (CT) and CT angiography revealed multiple active arterial bleeding within the subcutaneous tissue at the L5/S1 level, with a large hematoma measuring 18 × 11 cm and 6 cm in depth. Embolization was considered but ultimately not pursued by the interventional radiology team due to distal vessel involvement and a high risk of rupture (Fig. 1).

Initial laboratory work-up showed normal coagulation results and no other significant abnormalities. Tranexamic acid (1 g i.v.) was administered immediately after CT. Despite the detection of active arterial bleeding on CT angiography, interventional radiology advised against embolization due to the distal location of the extravasation and the considerable risk of vessel rupture in the context of mcEDS-associated vascular fragility. At the time of admission the patient was hemodynamically stable, reported only mild discomfort and had preserved hemoglobin levels. In light of these findings and the known surgical risks in mcEDS patients, including potential for tissue injury and anesthesia-related complications, a cautious, nonoperative approach with close observation on a surgical ward was initially favored.

This decision was guided by the desire to avoid unnecessary surgical trauma in a fragile patient if spontaneous hemostasis could be achieved.

Therefore, compression was applied using elastic bandages positioned over the lumbar swelling in an attempt to tamponade the bleeding. The patient was admitted to the surgical ward for close observation. Given the extent of the hematoma and the stable condition at the time, surgical evacuation was scheduled for the following day. The compression was reassessed clinically through repeated examinations and serial vital signs monitoring.

Despite bandaging, the swelling continued to expand, and the patient developed increasing discomfort. Only several hours later, at 2 a.m. the patient became pale and nauseated and showed signs of hypovolemic shock. The hemoglobin level dropped from 14.0 g/dL to 11.7 g/dL and the hematoma extended toward the scapular region, necessitating emergency surgical evacuation.

## Management and outcome

One of the initial challenges in the perioperative management was establishing peripheral venous access. Given the patient’s known vascular fragility and history of difficult cannulation, ultrasound guidance was employed enabling successful placement of a 20-gauge peripheral intravenous catheter.

Upon arrival in the operating theater the patient presented with significant pain, which rendered positioning in the supine position for induction intolerable. As a result, standard monitoring and preoxygenation were performed with the patient in the lateral decubitus position.

Anesthesia was induced using fentanyl, propofol, and rocuronium. Endotracheal intubation was successfully achieved in supine position via direct laryngoscopy without complications.

**Fig. 2:**
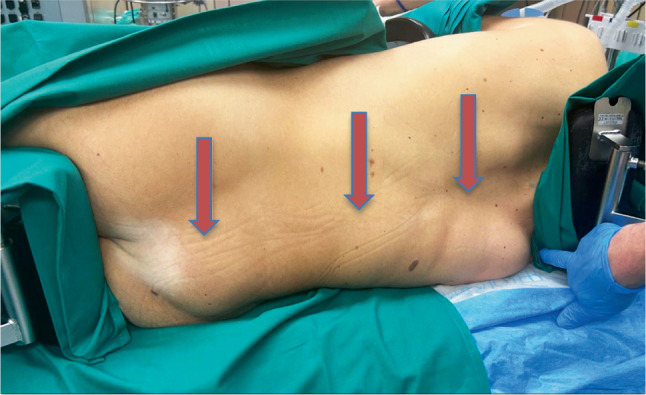
Intraoperative positioning of the patient. Notable progression of the hematoma over time with cranial extension reaching beneath the right scapula. The arrows indicate the extent of the hematoma, including cranial extension reaching beneath the right scapula

The patient was then positioned in the lateral decubitus position to facilitate exposure of the hematoma extending from the lumbar region toward the scapular area (Fig. 2).

However, shortly after induction, the patient developed progressive hemodynamic instability, requiring repeated bolus administration of vasopressors. Due to persistent hypotension, a continuous norepinephrine infusion was initiated, which effectively stabilized the patient’s circulatory status.

Surgical procedure: a 15 cm lumbar incision was made with the patient under general anesthesia. Subcutaneous tissue was detached from the fascia and filled with blood and coagula (more than 1.1 L). A second incision at the right scapular base released additional hematoma. Hemostasis was achieved using electrocautery and application of PerClot® (absorbable polysaccharide hemostatic powder, Baxter, Deerfield, IL, USA), which was distributed extensively into the wound cavity from the lumbar wound pockets to the scapular region, ensuring broad surface coverage.

The following intraoperative course proceeded uneventfully from both surgical and anesthetic perspectives. The patient received four units of packed red blood cells, 2 g fibrinogen and 20% human albumin to manage intraoperative blood loss and maintain hemodynamic stability. Due to ongoing difficulties with peripheral venous access and the patient requiring vasopressor support, a central venous catheter was placed under ultrasound guidance at the end of the procedure without complications.

Two surgical drains were inserted and subsequently removed on postoperative day 2, with no evidence of hematoma reaccumulation. The patient required two additional units of red blood cells up to postoperative day 3. Antibiotic therapy, initiated intraoperatively, was continued until suture removal.

**Fig. 3:**
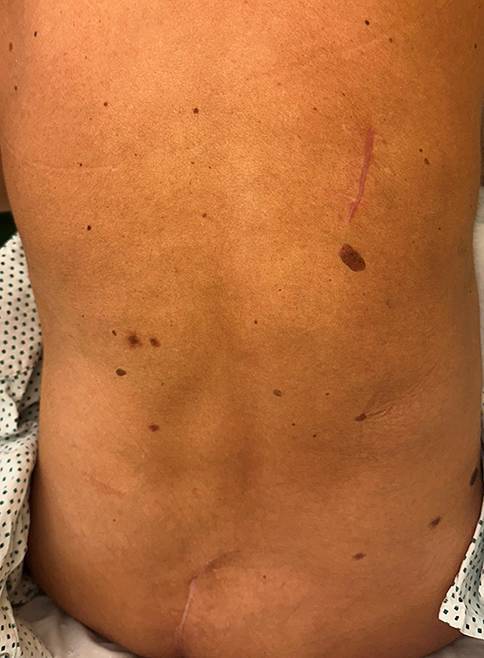
Postoperative situs after wound healing

Antibiotics were started intraoperatively and continued until suture removal. The patient was discharged in a stable condition on postoperative day 4. The sutures were removed on postoperative day 14. The local wound conditions remained stable, with no recurrent bleeding in the back region. Figure 3 shows the postoperative status approximately 1 month after surgical intervention.

## Discussion

Musculocontractural Ehlers-Danlos syndrome (mcEDS) is an extremely rare subtype of EDS, caused by pathogenic variants in CHST14 or *DSE* (dermatan sulfate epimerase, encoded by the DSE gene), leading to severe connective tissue fragility. One of the most serious complications in mcEDS is the formation of giant subcutaneous hematomas after minor trauma, which can result in progressive hemorrhagic shock. Management remains challenging due to the lack of established treatment protocols and the risk of iatrogenic injury.

A similar case was described by Uemura et al., involving a 6-year-old boy with mcEDS who developed hemorrhagic shock after a fall that caused a large subcutaneous hematoma in the left thigh. In their case, conservative management with limb elevation, compression, fluid resuscitation without red blood cell substitution, and tranexamic acid led to stabilization. Hemostasis was achieved nonsurgically, and the patient was discharged after 13 days of inpatient care [3].

In contrast, our case involved a young adult with mcEDS who developed a rapidly expanding hematoma in the lumbar region, progressing toward the scapula, accompanied by hemodynamic compromise. While initial conservative observation was considered, worsening clinical signs (pallor, hypoperfusion, decrease in hemoglobin levels) necessitated emergency surgical evacuation. In our case, embolization was not performed due to the anatomical characteristics of the bleeding vessels. The interventional radiology team assessed the case but decided against proceeding with embolization, citing the distal location of the arterial extravasation and a heightened risk of iatrogenic rupture, given the patient’s profound vessel fragility related to mcEDS. Consequently, surgical evacuation was selected as the most viable option once hematoma progression and early signs of circulatory compromise became evident. Hemostatic management was guided by clinical judgment and anticipated bleeding volume, as real-time viscoelastic testing was unavailable. We administered fibrinogen and human albumin to support coagulation and volume status. Additionally, we utilized extensive topical application of PerClot® to achieve localized hemostasis within fragile soft tissue cavities, thereby minimizing the need for further instrumentation or trauma. Antibiotic prophylaxis was initiated intraoperatively and continued until suture removal, in part due to the extensive subcutaneous wound cavities and the high risk of hematoma superinfection; however, we acknowledge that extended antibiotic courses increase the risk of nosocomial infections and are not routinely recommended in standard surgical practice.

This comparison highlights a crucial distinction: while conservative approaches may suffice in pediatric patients with localized hematomas and stable vital signs, adult patients with deep and expanding hemorrhages may require surgical management. Moreover, our case emphasizes the value of combining topical agents, systemic factor replacement and timely surgical intervention to avoid further decompensation.

## Conclusion

Patients with mcEDS require tailored trauma care due to a significant bleeding risk. Early surgical intervention may be lifesaving when hematoma expansion occurs. Multidisciplinary coordination and individualized hemostatic strategies are essential in managing such complex cases. Given the extreme tissue and vascular fragility in mcEDS, early recognition and tailored protocols are critical. One potential improvement in the management of such patients could be the implementation of an emergency medical passport, a compact document or digital tool summarizing the patient’s diagnosis, bleeding risks, prior interventions, preferred vascular access strategies, anesthetic considerations and contraindicated procedures. Such a tool could expedite multidisciplinary coordination and reduce delays or inappropriate interventions, particularly in emergency settings or in hospitals with limited experience managing rare connective tissue disorders.

## Data Availability

Data are available from the authors on reasonable request.
